# Association Analysis of Inter-Alpha-Trypsin Inhibitor Genes in Schizophrenia

**DOI:** 10.14789/ejmj.JMJ26-0008-OA

**Published:** 2026-06-05

**Authors:** WANYI MAO, TOHRU OHNUMA, SHOHEI NISHIMON, TAKAHIRO SANNOHE, NARIMASA KATSUTA, NOBUTO SHIBATA

**Affiliations:** 1 Department of Psychiatry and Behavioral Science, Juntendo University Graduate School of Medicine, Tokyo, Japan; 1 Department of Psychiatry and Behavioral Science, Juntendo University Graduate School of Medicine, Tokyo, Japan

**Keywords:** schizophrenia, genetic association, SNP, protein structure, risk factors

## Abstract

**Objectives:**

Schizophrenia (SCZ) is a common psychiatric disorder with relatively high morbidity and heritability. Affecting approximately 1% of the global population, SCZ is estimated to have an individual heritability of 60-85%. In recent years, several promising genetic loci have been identified using novel approaches such as genome-wide association studies (GWAS). Multiple GWAS have consistently reported that inter-alpha-trypsin inhibitor heavy chain (ITIH) family genes located on chromosome 3p21 are associated with SCZ in European populations, as demonstrated by the Schizophrenia Working Group of the Psychiatric Genomics Consortium and subsequent studies, as well as with bipolar disorder, another major psychotic disorder.

**Methods:**

In the present study, we conducted a case-control association analysis and haplotype analysis of single-nucleotide polymorphisms (SNPs) within the ITIH gene cluster at the 3p21 region to examine whether these variants confer genetic risk for schizophrenia in a Japanese population. In addition, we resequenced the ITIH1 gene to identify single-nucleotide variants (SNVs) and performed protein structural analyses to predict the effects of these variants on protein stability, aiming to clarify the potential contribution of ITIH1 to the pathophysiology of schizophrenia.

**Results:**

A significant difference in haplotype frequencies for the two-SNP window comprising rs2710322 and rs1042779 was observed. Resequencing identified 4 potentially deleterious variants.

**Conclusions:**

This study suggests a possible role of ITIH1 in the pathophysiology of schizophrenia.

## Introduction

Requiring long-term medical and social care, psychiatric disorders impose a substantial burden not only on patients and their families but also on society and healthcare systems. Schizophrenia (SCZ) is a common psychiatric disorder with relatively high morbidity and heritability^1)^. Affecting approximately 1% of the global population, SCZ is estimated to have an individual heritability of 60-85%. Because genetic factors are considered to play a central role in the etiology of psychiatric disorders, numerous genetic studies have been conducted to identify susceptibility genes for these conditions.

In recent years, several promising genetic loci have been identified using novel approaches, such as genome-wide association studies (GWAS). GWAS is a powerful tool for identifying common genetic risk factors underlying complex diseases. Several GWAS have consistently reported associations between schizophrenia and inter-alpha-trypsin inhibitor heavy chain (ITIH) family genes^1-5)^ located on chromosome 3p21, based on studies conducted in European populations by the Schizophrenia Working Group of the Psychiatric Genomics Consortium and subsequent investigations. These loci have also been implicated in bipolar affective disorder (BD), another major psychotic disorder.

Specifically, Ripke et al.^4)^ reported that rs2239547, located in the ITIH3-ITIH4 region, reached genome- wide significance (P = 7.8 × 10^−9^) in a joint GWAS involving 16,374 cases (patients with SCZ and BD) and 14,044 controls of European ancestry. Using joint analyses that incorporated SCZ data from the Psychiatric Genome-Wide Association Study Consortium, Hamshere et al. further confirmed that rs2239547 was significantly associated not only with SCZ (P = 3.62 × 10^−^^10^) but also with SCZ and BD in combined analyses. Another SNP in this region, rs2535627, was also reported to be associated with schizophrenia in European populations.

In Asian populations, GWAS data have shown that rs2239547 in ITIH4 is also a replicated association locus in Han Chinese patients with schizophrenia. Furthermore, a previous meta-analysis including both European and Asian populations (excluding Japanese individuals) demonstrated that rs2239547 in the ITIH3-ITIH4 region represents a genetic risk factor for schizophrenia. Clinically, rs2239547 has also been reported to be associated with suicide attempts in Norwegian patients with SCZ and BD^6-9)^.

In addition, an intronic SNP, rs2535629 in ITIH3, was identified as a genetic risk locus for schizophrenia by the European Psychiatric Genomics Consortium^5)^. This SNP was subsequently examined in a Japanese case-control study and was reported to confer genetic risk for schizophrenia, accompanied by altered peripheral ITIH4 gene expression levels^10)^. Moreover, this variant has been suggested to be associated with antipsychotic treatment response in European patients with schizophrenia^11)^.

Furthermore, an intronic SNP, rs2710322 in ITIH1, was reported to be significantly associated with schizophrenia in a Han Chinese population^12)^. Prior to this finding, rs1042779 in ITIH1 had been identified through GWAS and meta-analyses as a predictor of BD in European populations, supporting the hypothesis that common genetic factors may underlie multiple psychiatric disorders. In addition to their genetic associations, ITIH proteins are known to play important roles in inflammatory processes, a proposed pathophysiological mechanism of schizophrenia, as well as in carcinogenesis and metastasis.

These SNPs are located within a broad (~1 Mb) region of strong linkage disequilibrium encompassing multiple ITIH family genes. This extensive linkage disequilibrium complicates the identification of causative genes, as the associated SNPs are in linkage disequilibrium with more than 30 genes in the surrounding region. To date, however, no studies have performed comprehensive case-control association and haplotype analyses of these SNPs. Therefore, we conducted a case-control genetic association study and haplotype analysis of these SNPs within the ITIH gene family at the 3p21 region to determine whether they represent genetic risk factors for schizophrenia in a Japanese population.

## Materials and Methods

### Subjects

A case-control genetic association study was conducted using 608 unrelated Japanese patients with schizophrenia (304 males and 304 females; mean age, 40.2 ± 13.8 years) who met the DSM- IV criteria for schizophrenia based on structured clinical interviews, as well as 650 healthy control subjects (299 males and 351 females; mean age, 46.8 ± 15.3 years). All patients had no lifetime history of manic or depressive episodes. Healthy control subjects did not meet current or past criteria for any Axis I disorder as assessed by the Structured Clinical Interview for DSM-IV (SCID). All participants met the following criteria: (1) no systemic or neurological disease; (2) no history of head trauma with loss of consciousness; and (3) no lifetime history of alcohol or substance dependence. This study was approved by the Ethics Committee of the Juntendo University School of Medicine (approval number M01-0094), and all participants provided written informed consent prior to participation. All subjects were enrolled between November 2001 and March 2020.

For ITIH1 gene resequencing, a subset of 24 patients with schizophrenia (10 males and 14 females; mean age, 39 years) was selected, all of whom underwent the same psychiatric assessments described above.

### DNA extraction

**Figure 1:**
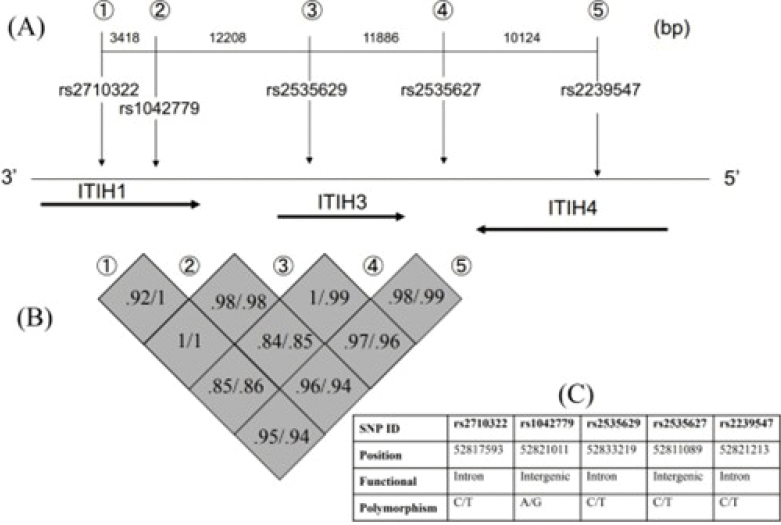
Genomic map of the human ITIH1, ITIH3, and ITIH4 genes showing the locations of the five SNPs (A) and the linkage disequilibrium (LD; D′ values) among SNPs within each gene (B). D′ values between SNP pairs are indicated within each diamond for control subjects (left) and patients with schizophrenia (right). Panel (C) summarizes information for the five SNPs.

Genomic DNA was extracted from peripheral blood leukocytes using a QIAamp^®^ DNA Blood Maxi Kit (Qiagen, Courtaboeuf, France). Five candidate SNPs within the ITIH gene family—rs2710322, rs1042779, rs2535629, rs2535627, and rs2239547—were selected based on previous GWAS, extension studies, and meta-analyses reporting positive associations with schizophrenia^2-12)^ (Figure 1).

### Genotyping

All selected SNPs were genotyped using TaqMan^®^ allelic discrimination assays (Assay-by-Design^TM^) on an ABI 7900 system (Applied Biosystems, Foster City, CA, USA). PCR reactions were performed using a standard PCR Master Mix reagent kit in a total reaction volume of 10 μL.

### Statistical analysis

Hardy-Weinberg equilibrium was assessed using SNPAlyse Ver. 7.0 Pro. Differences in genotypic and allelic frequencies between cases and controls were evaluated using chi-square (χ^2^) tests. Linkage disequilibrium (LD), expressed as D′, was estimated using an expectation-maximization algorithm, and LD blocks were defined as regions with D′ > 0.75. Case-control haplotype analyses were performed using SNPAlyse software, with permutation testing (10,000 replications) applied to obtain empirical P values. All statistical tests were two-tailed, and statistical significance was defined as P < 0.05. Power calculations were conducted using the Power Calculator for Two-Stage Association Studies.

### Sequencing, alignment, assembly

Twenty-four patients with schizophrenia were sequenced using Sanger sequencing. Reference sequences were obtained from the NCBI database (NM_001166434.3). Sequence alignment and assembly were performed using BWA (version 0.7.17-r1188) and SAMtools (version 1.7).

### Variant detection and annotation

Variant calling, including single-nucleotide polymorphisms (SNPs) and insertions/deletions (indels), was performed using GATK (version 4.0). Identified variants were annotated using ANNOVAR software.

### Prediction of protein structure

To assess the potential impact of missense variants on protein structure and stability, DUET was used as an integrated computational prediction tool. Structural comparisons between wild-type and mutant proteins were performed using iCn3D. Functional effects of amino acid substitutions were further evaluated using SIFT, with variants having normalized probabilities < 0.05 considered deleterious.

## Results

The mean age did not differ significantly between the schizophrenia group and the control group (p > 0.05), and there was no significant difference in sex distribution between the two groups. No deviations from Hardy-Weinberg equilibrium (HWE) were observed for any of the five SNPs examined in the Japanese subjects (Table 1).

**Table 1:** Distribution and statistical analysis of the *ITIH1*, *ITIH3* and *ITIH4* gene polymorphisms

	Genotype frequency (%)			*P*-value	HWE	Allele frequency (%)		χ^2^	*P*-value	Odds ratio (95%CI)
*ITIH1*										
rs2710322	C/C	C/T	T/T			C	T			0.998
schizophrenia	402 (56.9)	268 (37.9)	37 (5.2)	0.961	0.352	1072 (75.8)	342 (24.2)	6.132E-4	0.980	(0.842-1.183)
controls	424 (57.1)	278 (37.4)	41 (5.5)			1126 (75.8)	360 (24.2)			
rs1042779	G/G	A/G	A/A			G	A			0.936
schizophrenia	183 (28.0)	307 (46.9)	164 (25.1)	0.137	0.789	673 (51.5)	635 (48.5)	0.434	0.51	(0.807-1.086)
controls	179 (24.2)	385 (52.0)	176 (23.8)			737 (49.8)	743 (50.2)			
*ITIH3*										
rs2535629	G/G	G/A	A/A			G	A			0.991
schizophrenia	231 (31.8)	355 (48.9)	140 (19.3)	0.624	0.489	817 (56.3)	635 (43.7)	0.016	0.8998	(0.856-1.147)
controls	221 (30.3)	375 (51.4)	133 (18.2)			817 (56.0)	641 (44.0)			
rs2535627	C/C	C/T	T/T			C	T			0.995
schizophrenia	199 (26.8)	353 (47.6)	190 (25.6)	0.061	0.675	751 (50.6)	733 (49.4)	4.035E-3	0.949	(0.861-1.151)
controls	169 (23.6)	385 (53.8)	162 (22.6)			723 (50.5)	709 (49.5)			
*ITIH4*										
rs2239547	T/T	T/C	C/C			T	C			0.913
schizophrenia	294 (39.8)	369 (50.0)	75 (10.2)	0.046	0.140	957 (64.8)	519 (35.2)	1.411	0.235	(0.785-1.061)
controls	287 (39.7)	333 (46.1)	103 (14.2)			907 (62.7)	539 (37.3)			

HWE; global *P*-value from Hardy-Weinberg equilibrium

Genotype and allele frequency distributions are summarized in Table 1. The genotypic distribution of rs2239547 in ITIH4 showed a marginally significant difference between patients with schizophrenia and controls (p = 0.046); however, this association did not remain significant after Bonferroni correction (adjusted significance threshold, p < 0.016). No significant differences were observed in either genotypic or allelic frequencies for the remaining four SNPs examined.

Case-control haplotype analyses using sliding windows of two to five SNPs revealed a significant difference in haplotype frequencies for the two-SNP window comprising rs2710322 and rs1042779 (global p = 0.007; Table 2). Notably, although neither rs2710322 nor rs1042779 showed a significant individual association with schizophrenia, their combined two-SNP haplotype was significantly associated with schizophrenia in the Japanese population. Because these two SNPs were in strong linkage disequilibrium (LD) in both patient and control groups (Figure 1B), this approximately 3-kb region may harbor Japanese-specific risk variants for schizophrenia.

**Table 2:** Case-control haplotype analysis from 2-windows to 5-windows

	2SNP-based				3SNP-based			4SNP-based		5SNP-based
rs2710322										
	**0.007**									
rs1042779					0.148					
		0.873						0.253		
rs2535629						0.975				0.124
			0.792						0.902	
rs2535627							0.458			
				0.305						
rs2238547										

Data shown are global *P*-value. Corrected *P* < 0.01 (0.05/5) is in bold.

Resequencing analysis of the ITIH1 gene identified a total of eight variant loci, including seven single-nucleotide polymorphisms (SNPs) and one insertion/deletion (indel), with four variants located in intronic regions and four in exonic regions (Table 3). An intronic variant at position 13276 was detected in all 24 sequenced samples.

**Figure 2:**
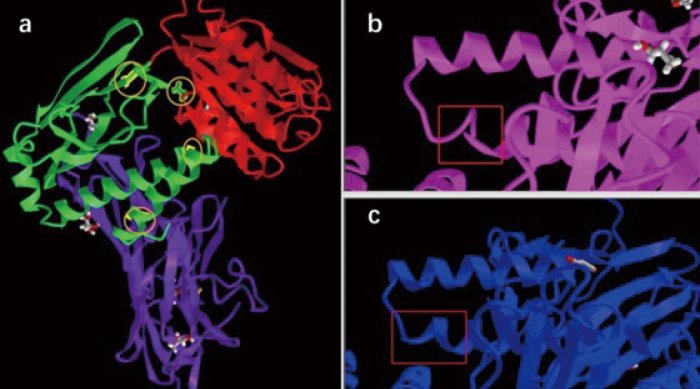
Structure of the ITIH1 protein (a) Locations of exonic mutations mapped onto the protein structure; (b) wild-type protein structure; (c) mutant protein structure.

**Figure 3:**
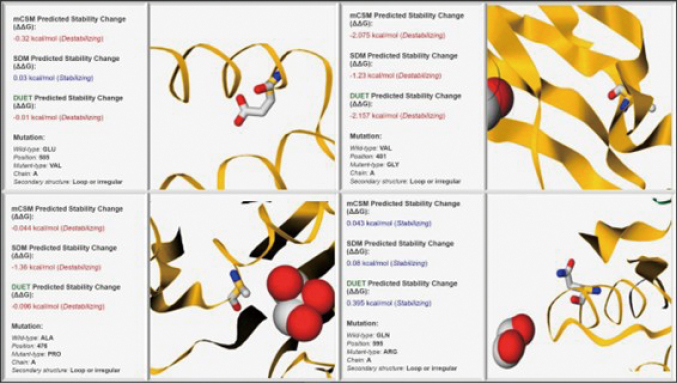
Predicted protein structures of the four exonic variants and assessment of their effects on protein stability

All four exonic variants were annotated as nonsynonymous SNVs. Their positions were mapped onto the corresponding protein structures, and structural differences between wild-type and mutant proteins were identified (Figure 2). Stability prediction using DUET indicated that the variant at position 14404 did not cause a substantial change in protein stability, whereas variants at positions 12471, 12487, and 14374 were predicted to result in marked structural instability (Figure 3). Consistent with these findings, SIFT analysis predicted these variants to be deleterious, with damaging scores of 0.985, 0.936, and 0.998, respectively.

**Table 3:** Variation and annotation results of the ITIH1 gene

Chr	Start	End	Ref	Alt	Func.refGene	AAChange.refGene	ExonicFunc.refGene	rate
NG_016005.1	10986	10986	T	C	intronic		.	0.375
NG_016005.1	11972	11972	G	A	intronic		.	0.625
NG_016005.1	12471	12471	G	C	exonic	exon12:c.G1426C:p.A476P	nonsynonymous SNV	0.125
NG_016005.1	12487	12487	T	G	exonic	exon12:c.T1442G:p.V481G	nonsynonymous SNV	0.125
NG_016005.1	13276	13276	T	G	intronic		.	1
NG_016005.1	14249	14252	GAAT	-	intronic		.	0.25
NG_016005.1	14374	14374	A	T	exonic	exon14:c.A1754T:p.E585V	nonsynonymous SNV	0.125
NG_016005.1	14404	14404	A	G	exonic	exon14:c.A1784G:p.Q595R	nonsynonymous SNV	0.25

**Figure 4:**
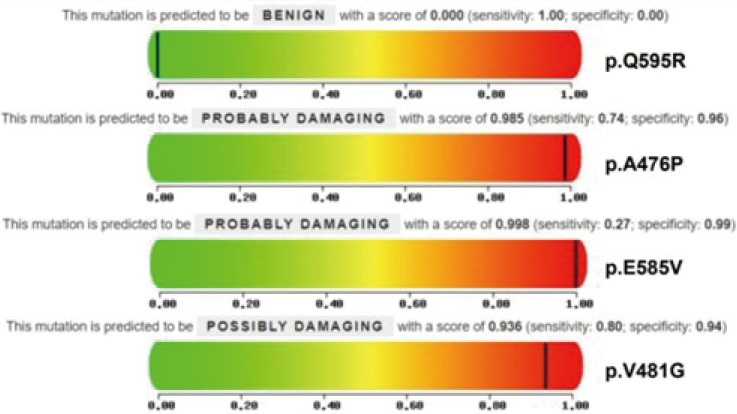
Predicted functional impact of each variant

## Discussion

In the present study, we re-examined whether previously reported associations between common SNPs within the ITIH1-ITIH3-ITIH4 genomic region and schizophrenia could also be observed in a Japanese population, and we further investigated the association between ITIH1 genetic polymorphisms and the risk of schizophrenia. The present results indicated that no single SNP examined conferred a significant genetic risk for schizophrenia in the Japanese population. In contrast, these SNPs have been associated with an increased risk of schizophrenia in GWAS conducted in other populations. These findings suggest the presence of ethnic differences between Japanese and other populations with respect to the genetic risk of schizophrenia, consistent with previous reports of substantial ethnic variation in metabolic gene polymorphisms^13)^.

Previous studies have reported significant haplotype and locus heterogeneity; however, most association signals have not reached genome-wide significance, thereby further rejecting the hypothesis of a single major risk gene for schizophrenia. Based on these observations, schizophrenia can be considered a polygenic disorder, with susceptibility arising from the cumulative effects of numerous genetic variants with small effect sizes, ranging from single-nucleotide variants (SNVs) and small insertions/deletions (indels) to copy number variations (CNVs) and large structural variants (SVs).

In addition, we performed resequencing of the ITIH1 gene followed by subsequent analyses. This gene encodes a member of the inter-alpha-trypsin inhibitor family of proteins, for which alternative splicing results in multiple transcript variants^14)^. At least one transcript encodes a preproprotein that is proteolytically processed to generate the heavy chain of the inter-alpha-trypsin inhibitor complex, which is secreted by hepatocytes into the circulation. This protein has been implicated in ovulation and fertilization and plays important roles in inflammatory processes.

In the context of our study, exonic mutations in ITIH1 were shown to have a pronounced impact on protein structural stability and were predicted to be deleterious. Furthermore, a study by Eum et al.^15)^ reported that analyses accounting for anticholinergic exposure from both psychiatric and non- psychiatric medications identified five significantly associated variants at the chromosome 3p21.1 locus, with the strongest association observed for rs1076425 in the ITIH1 gene (P = 3.25 × 10^-9^).

This study has several limitations that should be considered when interpreting the results. In particular, the number of samples included in the ITIH1 resequencing analysis (n = 24) was too small to draw any definite conclusions.

In conclusion, our findings suggest that ITIH1 might be associated with the risk of schizophrenia in the Japanese population. We also demonstrated that exonic variants at three loci lead to structural instability and predicted deleteriousness of the ITIH1 protein. Overall, this study provides preliminary genetic evidence supporting a possible role for ITIH1 in the pathophysiology of schizophrenia.

## Data availability

The data are available upon request.

## Author contributions

TO conceived the project. WM performed genetic analyses and wrote the paper. TO, SN, TS, NK, and NS collected samples.

## Conflicts of interest statement

The authors declare that there are no conflicts of interest.
